# Nonlinear data fusion over Entity–Relation graphs for Drug–Target Interaction prediction

**DOI:** 10.1093/bioinformatics/btad348

**Published:** 2023-05-31

**Authors:** Eugenio Mazzone, Yves Moreau, Piero Fariselli, Daniele Raimondi

**Affiliations:** Department of Medical Sciences, University of Torino, 10123 Torino, Italy; Department of Electrical Engineering, ESAT-STADIUS, KU Leuven, 3001 Leuven, Belgium; Department of Medical Sciences, University of Torino, 10123 Torino, Italy; Department of Electrical Engineering, ESAT-STADIUS, KU Leuven, 3001 Leuven, Belgium

## Abstract

**Motivation:**

The prediction of reliable Drug–Target Interactions (DTIs) is a key task in computer-aided drug design and repurposing. Here, we present a new approach based on data fusion for DTI prediction built on top of the NXTfusion library, which generalizes the Matrix Factorization paradigm by extending it to the nonlinear inference over Entity–Relation graphs.

**Results:**

We benchmarked our approach on five datasets and we compared our models against state-of-the-art methods. Our models outperform most of the existing methods and, simultaneously, retain the flexibility to predict both DTIs as binary classification and regression of the real-valued drug–target affinity, competing with models built explicitly for each task. Moreover, our findings suggest that the validation of DTI methods should be stricter than what has been proposed in some previous studies, focusing more on mimicking real-life DTI settings where predictions for previously unseen drugs, proteins, and drug–protein pairs are needed. These settings are exactly the context in which the benefit of integrating heterogeneous information with our Entity–Relation data fusion approach is the most evident.

**Availability and implementation:**

All software and data are available at https://github.com/eugeniomazzone/CPI-NXTFusion and https://pypi.org/project/NXTfusion/.

## 1 Introduction

*In silico* methods for the discovery of Drug–Target Interactions (DTIs) are important to speed up drug discovery and drug repurposing (Öztürk *et al.* 2018), which are expensive and time-consuming experimental endeavors because of the immensity of the space of possible compounds (Whitebread *et al.* 2005, Huang *et al.* 2021).

Several DTI prediction methods have been developed so far, based on strategies, such as docking (Morris *et al.* 2009), classical Machine Learning (ML) (Pahikkala *et al.* 2015, He *et al.* 2017), or deep learning (Wen *et al.* 2017, Öztürk *et al.* 2018, Tsubaki *et al.* 2019). Another family of approaches relies on the “guilt by association” principle (Luo *et al.* 2017), which is based on the observation that similar chemical compounds tend to bind to similar proteins (targets), and *vice-versa*. Recommender-like systems based on matrix factorization (MF) have been developed for DTI prediction (Zheng *et al.* 2013, Arany *et al.* 2015) following this principle.

DTIs databases, such as DrugBank (Law *et al.* 2014) and ChEMBL (Bento *et al.* 2014), contain from thousands to millions of DTIs. Still, the DTI space is so vast that frequently only a limited number of interactions are known for each protein, thus impairing generalization in classical ML methods. For this reason, it has become important for DTI predictors to incorporate heterogeneous sources of information to contextualize different molecular, genomics, medical, and chemical aspects of both drugs and proteins, improving their generalization potential (Luo *et al.* 2017). For example, methods integrating the side effects of drugs (Campillos *et al.* 2008), drug–disease associations (Wang *et al.* 2014, Luo *et al.* 2017), and gene expression (Sirota *et al.* 2011) have been developed.

Data fusion methods allow the integration of heterogeneous sources of information, thereby providing bioinformatics models with a sufficiently multi-faceted knowledge to increase their generalization ability. They have already demonstrated their usefulness in tackling relevant bioinformatics problems (Aerts *et al.* 2006, Sifrim *et al.* 2013, Raimondi *et al.* 2021). Recently, we developed a Neural Network-based scalable data fusion library, called NXTfusion (Raimondi *et al.* 2021), which generalizes the MF-based data fusion paradigm by extending it to the nonlinear inference over Entity–Relation (ER) graphs. NXTfusion represents arbitrarily connected heterogeneous data as Relations (e.g. sparsely observed matrices) connecting classes of objects (Entities). The resulting ER graphs are conceptually similar to a relational database on which inference can be globally performed through multi-task learning. To do so, NXTfusion internally transforms the inference over abstract ER graphs into the concurrent (multitask) nonlinear factorization of the observed data matrices (relations).

In this article, we leverage our NXTfusion library to build several dataset-specific DTI predictors and benchmark them against state-of-the-art methods. We exploit the flexibility of our ER graph formalism to show the benefits of the integration of medical, biological, and chemical heterogeneous sources of information through nonlinear data fusion in terms of generalization ability. We trained and tested our model on five publicly available datasets, showing that our models (i) outperform the state-of-the-art on several of them and (ii) are flexible enough to predict both DTIs as binary classification and regression of the real-valued drug–target affinity, competing with models specifically built for this task.

Moreover, we show that the validation performed by some state-of-the-art approaches could be made stricter and thus more realistic, in line with the previous findings from Pahikkala *et al.* (2015). In real-life settings, DTI predictions could be needed for drugs or proteins for which no already known DTIs are available. These stricter settings are exactly the context in which the benefit from the integration of heterogeneous information with our ER data fusion approach is most visible.

## 2 Materials and methods

### 2.1 Dataset

We used five datasets from literature to train, test, and validate our model, comparing it with state-of-the-art approaches.

*LHU and LCE datasets*: We retrieved two datasets from Tsubaki *et al.* (2019). They were originally proposed by Liu *et al.* (2015) and they respectively contain DTIs from *Caenorhabditis elegans* and human. We refer to these datasets as LHU (Liu HUman) and LCE (Liu *C.elegans*) from now on. As described in Liu *et al.* (2015), the peculiarity of these datasets is that the noninteracting drug–protein pairs are obtained by creating highly credible negative samples, based on the assumption that proteins that are dissimilar to any known target of a given compound *C* are not likely to be targeted by *C* and *vice-versa* (Liu *et al.* 2015). Positive samples were retrieved from DrugBank 4.1 (Wishart *et al.* 2008) and Matador (Günther *et al.* 2007). The human dataset (LHU) contains 3364 positive DTIs between 1179 unique compounds and 834 unique proteins; the *C.Elegans* dataset (LCE) contains 3893 positive interactions between 968 unique compounds and 814 unique proteins.

*YUNAN dataset*: We also retrieved the dataset used in Luo *et al.* (2017) and refer to it as YUNAN in the rest of the article. YUNAN contains a total of 1493 proteins and 708 different drugs. The known DTIs were extracted from DrugBank 3.0 (Knox *et al.* 2011). The authors also provide additional information related to the self-interaction of both proteins and drugs, and we retrieved them as well. Protein–Protein Interactions (PPI) came from HPRD 9.0 (Keshava Prasad *et al.* 2009) and Drug–Drug Interactions (DDI) from DrugBank 3.0. We also retrieved data on protein–protein similarity, drug–drug similarity, protein–disease, and drug–disease associations from the Comparative Toxicogenomics Database (Davis *et al.* 2013) and drug–side effect data from SIDER database 2.0 (Kuhn *et al.* 2010).

*Kinase datasets*: We also benchmarked our work on two different kinase datasets, Davis (Davis *et al.* 2011) and KiBA (Tang *et al.* 2014), which were previously used as benchmark datasets for binding affinity prediction evaluation (Pahikkala *et al.* 2015, He *et al.* 2017). The Davis dataset contains selectivity assays of the kinase protein family and the relevant inhibitors with their respective dissociation constant (*K*d) values. It comprises interactions of 442 proteins and 68 ligands. The KiBA dataset, by contrast, originated from an approach called KiBA (Tang *et al.* 2014), in which kinase inhibitor bioactivities from different sources, such as Ki, Kd, and IC50, were combined. The KiBA dataset originally comprised 467 targets and 52 498 drugs. In order to ensure reproducibility and fair comparison with recent methods benchmarked on KiBA, we used dataset version proposed in He *et al.* (2017), where the authors filtered it to contain only drugs and targets with at least 10 interactions yielding a total of 229 unique proteins and 2111 unique drugs. Similarly to He *et al.* (2017) and Öztürk *et al.* (2018), we transformed the *K*d values into log spaceto obtain the final regression labels.

### 2.2 The NXTfusion framework for nonlinear ER data fusion

To construct our DTI prediction models, we used NXTfusion, a PyTorch (Paszke *et al.* 2017) framework for ER data fusion. We briefly recapitulate its principles here, referring the reader to Raimondi *et al.* (2021) for more details.

In the classical MF data fusion paradigm (Mnih and Salakhutdinov 2008, Arany *et al.* 2015, Simm *et al.* 2015, Žitnik and Zupan 2015), a target matrix (relation) Y=UV is reconstructed by the product of two rectangular matrices *U*, *V*, such that where $||\cdot |{|}_{F}$ is the Frobenius norm and λ the regularization weight. In this way, *U* and *V* are optimized to respectively containing a latent representations of the objects (entities) listed in the rows (*U*) and columns (*V*) of *Y*. Each matrix *Y* thus represents a “relation” between the “entities” listed as elements in the rows and columns. NXTfusion extends this classical MF paradigm, in which a single matrix is factorized, allowing (i) a nonlinear relationship between *U* and *V* and (ii) the concurrent factorization of an arbitrary number of matrices (now called Relations) between an arbitrary number of pairs Entities (the row and columns of each matrix) (Raimondi *et al.* 2021).


$$\underset{U,V}{\text{argmin}}||Y-UV|{|}_{F}+\lambda (||U|{|}_{F}+||V|{|}_{F}),$$

**Figure 1.**
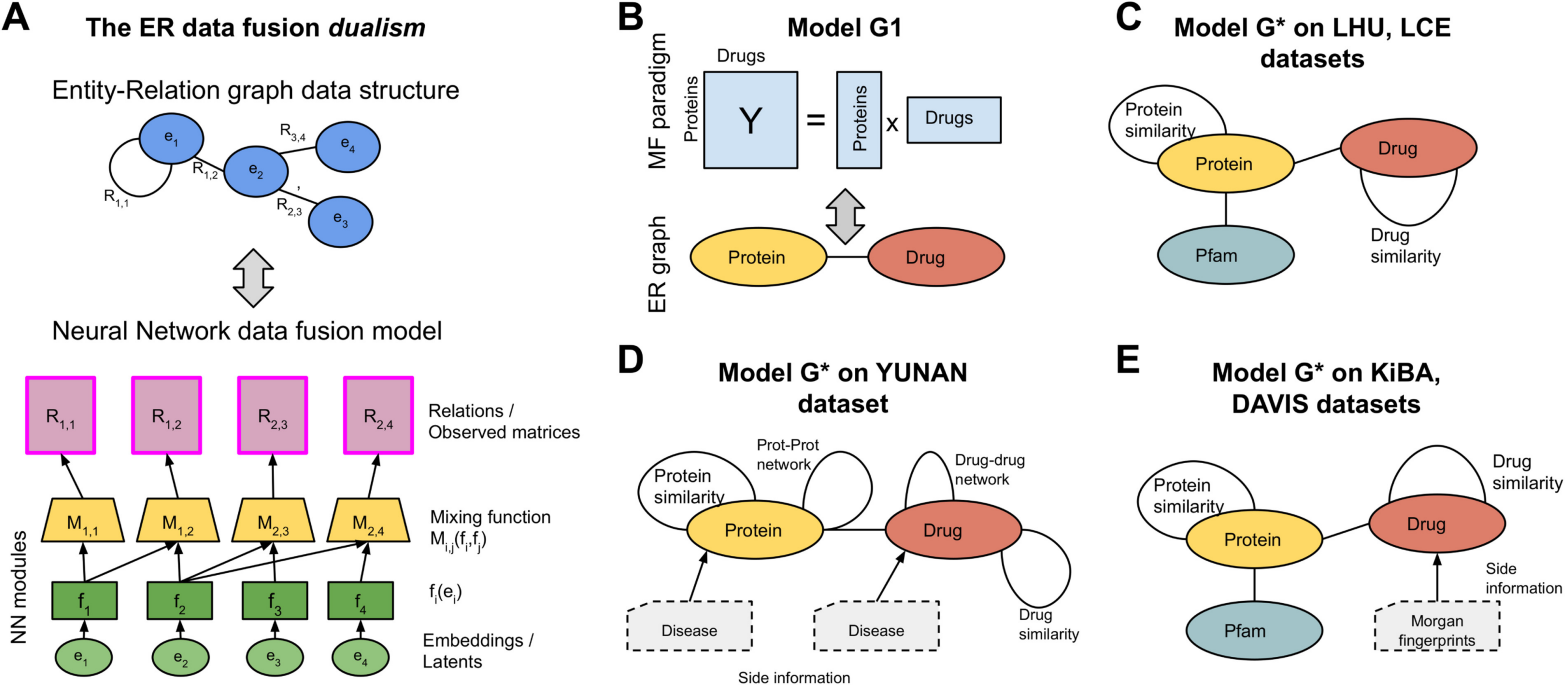
Overview figure. Panel A shows the dualism between the ER graph describing the conceptual organization of the data (top) and the NN architecture used to perform inference on the ER graph (bottom). Panel B shows the baseline G1 model, in which only the DTI relation between the drug and protein entity modeled. Panels C, D, and E show the dataset-specific instances of ER graphs, we used to build the G* models in each of the five benchmark datasets.

As shown in Fig. 1A, NXTfusion detaches the abstract data fusion problem from the low-level details of the MF by representing arbitrarily complex and heterogeneous data collections as abstract ER graphs, where circles represent Entities and edges represent Relations (data matrices) connecting them, allowing high-level reasoning on the data and their interactions. The low-level “dual” representation of the ER graph, used to perform inference, is a problem-specific Neural Network (NN) architecture (see lower half of Fig. 1A), whose details are automatically managed by the NXTfusion library, allowing the user to reason in terms of abstract ER graphs (upper half of Fig. 1A).

The library represents each Entity $E_{i}$ (Fig. 1A) with embeddings $e_{i}$ (i.e. trainable parameters). These $e_{i}$ are then transformed by an entity-specific module (i.e. some NN layers) $f_{i}(e_{i})$. Two entities $E_{i},E_{j}$ participating in a relation $R_{ij}$ are thus the input of the relation-specific module $M_{ij}$, which consists of a bilinear layer followed by a feed-forward (FF) layer. As shown in Fig. 1A, for each $R_{ij}$, the corresponding mixing function $M_{ij}$ produces the predicted outputs.

The ER model is thus globally optimized to minimize, for each $R_{ij}$, a relation-specific loss


$$L_{ij}=L_{ij}(R_{ij},M_{ij}(f_{i}(e_{i}),f_{j}(e_{j}))).$$

The final objective function to minimize with respect to the NN parameters for a given ER graph G is thus which allows for all the relations (matrices) to be learned concurrently, weighted by the task-specific scale factor $\omega_{ij}$. The NN architecture underlying the ER data fusion problem is implemented in PyTorch, and the inference is thus equivalent to training a FF multitask NN. This dualism is illustrated in Fig. 1A. The fact that each relation $R_{ij}$ has a specific loss $L_{ij}$ allows the NXTfusion data fusion framework to be flexible in incorporating any kind of relation, independently from the type of prediction problem it presents (e.g. binary classification, regression, or multiclass prediction). The scale factor $\omega_{ij}$ is needed to balance the absolute values of the losses, which can differ significantly between tasks, depending on the size of the matrices, the magnitude of their values, their sparsity, and the chosen loss functions (Raimondi *et al.* 2021). In this study, we assigned a uniform relevance $\omega_{ij}$ to all the relations.


$$\sum_{\forall R_{ij}\in G}\omega_{ij}L_{ij},$$

The key intuition behind data fusion through multitask prediction (factorization) of tasks (relations) organized as ER graphs is that adding “auxiliary” tasks (relations) to be learned alongside the main task (DTI prediction in this case) could introduce additional information in the inference process if these auxiliary tasks are carefully chosen (Raimondi *et al.* 2021). Forcing the model to learn additional aspects involving the entities of the ER graph could indeed push the model toward learning a richer latent embedding representation compared to learning only to reconstruct the main relation.

The NXTfusion library is available as a Python package at https://pypi.org/project/NXTfusion/.

### 2.3 ER data fusion models for DTI prediction

In this article, we use five datasets to train and test our ER data fusion models. Each dataset presents an instance of the DTI prediction problem, but they all have certain differences in terms of the types of “contextual” data available. In Fig. 1B–E, we showcase the variety of dataset-specific models that can be built with the NXTfusion framework by devising a specific ER graph for each dataset, including all the contextual information available in each case.

For each dataset, we used as a “baseline” the model representing the simplest ER graph possible, in which only the main task (the DTI relation between drug and target proteins) is considered (see Fig. 1B). This is analogous to the classical Y=UV setting in MF, except that the $f_{i}$ and the *M_ij_* are nonlinear functions. We refer to this simple ER graph as G1 (see Fig. 1B). For each dataset, we then compare G1 to more extensive data fusion models over arbitrary ER graphs (called G*), where multiple relations between multiple entities are modeled (i.e. protein–Pfam domains, drug–disease), encompassing all the dataset-specific contextual data available (see Fig. 1B–E). Here, we summarize the additional relations used to build the G* models for each dataset:

*LHU and LEC datasets*: As shown in Fig. 1C, the ER graph for the LHU and LEC datasets contains several additional relations compared to the simple G1 model (Fig. 1B). We added a self-relation on the protein entity representing the protein similarity computed with BLAST (Altschul *et al.* 1990). We represented the protein–protein similarity as a matrix containing the bit score outputted by BLAST normalized by row. We also added a drug–drug self-relation representing the similarity between drugs, computed with RDkit (Landrum 2013) from Morgan fingerprints (radius =2, nBits = 1028). This is a dense matrix where every drug pair is associated with a continuous value between 0 and 1. We considered both of these auxiliary tasks as regressions, using a mean squared error (MSE) loss function. We also used PfamScan (Mistry *et al.* 2007) to retrieve all the Pfam (Finn *et al.* 2014) domains for the target proteins. For each protein, we thus built a binary matrix listing the Pfam domains. We tried it both as additional protein–domain relation and as side information (i.e. classical ML features). In the latter case, we mixed these features with the protein embedding via a bilinear layer.

*YUNAN dataset*: Besides the main DTI task, four additional matrices were provided by the authors of Luo *et al.* (2017). We added them as auxiliary Relations in our G* ER models (see Fig. 1D). Two of them are self-relations representing respectively the similarity between proteins and between drugs. The other two relations represent PPI and DDI networks. We integrated these four auxiliary relations in our G* ER model as regression problems. Last, we added the Pfam-domain annotations retrieved with PfamScan (Mistry *et al.* 2007). As described previously, we tried it both as side information or additional task (see Section 3).

*KiBA and Davis datasets*: We added the same relations described for LHU and LEC datasets, namely the self-relations describing the protein–protein and the drug–drug similarity (computed respectively with BLAST and RDKit) and the protein–domain relation using PfamScan annotations as both side information and additional task.

Moreover, we computed Morgan fingerprints with 30 000 dimensions (Morgan 1965) for each drugs in KiBA and DAVIS using the RDkit (https://www.rdkit.org/) library. These fingerprints are highly sparse binary representation of drugs (only 0.1%–0.2% of 1 s), and we added them as sparse side information, to minimize the computational overhead. The G* ER graph, we used on KiBA and Davis is shown in Fig. 1E.

### 2.4 Implementation

We used the Binary Cross Entropy loss function on the LHU, LCE, and YUNAN datasets, with class balance parameter equal to the positive to negative label ratio. On KiBA and DAVIS datasets, we used the MSE loss.

To train the models, we used the Adam optimizer, with learning rate 0.001 and weight decay 0.001. In the $f_{i}$ and $M_{ij}$ modules, we used Tanh activations, preceded by layer normalization (Ba *et al.* 2016). The $f_{i}$ is a FF NN with 3 layers and 10 neurons for the drug latent representation and 20 neurons for the protein latent representation. The $M_{ij}$ module is an FF NN with 2 layers and 10 neurons. The protein, drug, and Pfam-domain embeddings have respectively 30, 20, and 10 dimensions. We did not perform hyper-parameter optimization, using the default parameters provided by the NXTfusion library.

The code to reproduce the experiments shown here is available at https://github.com/eugeniomazzone/CPI-NXTFusion.

## 3 Results

### 3.1 Nonlinear data fusion improves over the state-of-the-art on the LCE and LHU datasets

We first benchmarked our G1 data fusion model, namely the nonlinear factorization of a single relation between the protein and drug entities (see Section 2 and Fig. 1B), on the *C.elegans* (LCE) and Human (LHU) datasets from Liu *et al.* (2015). LCE and LHU are balanced datasets with a ratio between positive samples (interacting protein–drug pairs) and negative samples (not-interacting pairs) of 1:1. To ensure a fair comparison with the other methods benchmarked on LCE and LHU, such as the Graph Convolutional NN (GCNN) proposed in Tsubaki *et al.* (2019), we reproduced the same 5-fold cross-validation (CV), where the pairs in each fold were randomly selected.

Tables 1 and 2 show the benchmark on the LEC and LHU datasets. We report the performance of the *k*-Nearest Neighbors (*k*-NN), Random Forest (RF), Logistic Regression (LR), Support Vector Machine (SVM), and the GCNN as presented in Tsubaki *et al.* (2019). We evaluated the prediction performance with the Area Under the ROC Curve (AUC), Area Under the Precision–Recall Curve (AUPRC), and the precision and recall measures.

**Table 1.** Benchmark of predictors on the LHU dataset.^a^

Model	AUC	AUPR	Precision	Recall
*k*-NN	0.860		0.798	0.927
RF	0.940		0.861	0.897
LR	0.911		0.891	0.913
SVM	0.910		0.966	0.950
GCNN	0.970		0.923	0.918

G1	0.992	0.988	0.975	0.970

a Scores are reported from Tsubaki *et al.* (2019), except for our G1 data fusion model.

**Table 2.** Benchmark of predictors on the LCE dataset^a^.

Model	AUC	AUPR	Precision	Recall
*k*-NN	0.858		0.801	0.827
RF	0.902		0.821	0.844
LR	0.892		0.890	0.877
SVM	0.894		0.785	0.818
GCNN	0.978		0.938	0.929

G1	0.990	0.992	0.911	0.989

a Scores are reported from Tsubaki *et al.* (2019), except for our G1 data fusion model.

Our G1 model performs 2%–3% higher than the GCNN from Tsubaki *et al.* (2019) on LCE and 2% higher on LHU. However, on these datasets, AUCs are generally high for most methods, including conventional ML methods, such as SVMs.

### 3.2 The stratification of the CV folds drastically influences the prediction performance on the LCE and LHU datasets

The DTI prediction problem is complex, and to be definitively solved, it requires to model nontrivial molecular aspects, such as the chemical and structural determinants of protein–drug binding affinity. An extensive study on the factors that can lead to over-optimistic DTI prediction results has been previously published in Pahikkala *et al.* (2015), and indeed also the high prediction performances shown in Tables 1 and 2 are likely to be caused by spurious effects, such as limits of the validation procedure used. We thus investigate these results by devising a more stringent CV mimicking the realistic scenario in which the model is required to predict drug or protein entities for which no information is available in the training set, in line with the recommendations for more stringent CV settings proposed in Pahikkala *et al.* (2015). In Table 3, we thus benchmarked our G1 approach in the following increasingly challenging scenarios on the LHU dataset:

**Table 3.** Comparison of the performance obtained by the G1 model with increasingly stringent CV stratification settings on the LHU dataset.

CV type	AUC	AUPR	Precision	Recall
Random folds	0.992	0.991	0.975	0.970
No stratification	0.999	0.995	0.994	0.993
Protein stratification	0.995	0.897	0.993	0.978
Drug stratification	0.915	0.879	0.821	0.736
Pair stratification	0.501	0.538	0.100	0.222

1. *Random folds:* CV folds are randomly selected, as done in Tsubaki *et al.* (2019) and Liu *et al.* (2015). For further details, see Supplementary Tables S1 and S2.
2. *No stratification:* Each protein and drug in the test set appears at least once in the training set.
3. *Protein stratification:* The proteins that occur in the training sets are not present in the test set. For nonfurther details, see Supplementary Tables S3 and S4.
4. *Drug stratification:* The drugs that appear in the training folds do not appear in the test sets. For further details, see Supplementary Tables S5 and S6.
5. *Pair stratification:* We stratified the CV folds ensuring that “both” proteins and drugs that appear in the test set were not present in the training folds. This is the most stringent setting.

Table 3 shows that the best performance is obtained in the no-stratification setting, because the model observes at least one data point for each protein and drug during training. The random-fold performance is similar but slightly lower, since there is no guarantee that each protein or drug in the test has been already observed during training. Surprisingly, the performance of both protein and drug stratification is still quite high, even if our G1 model (see Fig. 1B) is just a nonlinear factorization of the protein–drug relation, and thus it has no way of modeling completely unseen instances of the protein or drug entities.

We thus investigate this behavior further and we found that it might be explained by the fact that in the LHU dataset, only a fraction (6%) of the protein and drug instances are involved in both positive and negative interactions, while the majority of them (94%) has only positive or negative interactions (see Supplementary Table S7). The surprisingly high performance of G1 on the protein and drug stratifications can thus be explained by the fact that the model uses the nonstratified entity to learn whether a certain drug or protein is “generally active” or “inactive” in the LHU dataset, independently of the other protein or drug partner in the interaction. This behavior allows the model to “bypass” the actual DTI task by making the prediction trivial.

The pair-stratification setting, in which our G1 model is required to predict never-seen-before (protein and drug) pairs, is the most stringent CV setting, and indeed the single-relation G1 model cannot perform better than random, since it has no way to model the latent representations of unseen entities instances.

### 3.3 Extending the G1 model with auxiliary relations provides additional information

Because of the peculiar distribution of the positives and negative cases in the LHU dataset shown in the previous section, we consider the pair-stratification setting the most meaningful performance evaluation strategy on this data, and we use this setting henceforth.

In this stratification setting, the G1 model cannot meaningfully predict the DTIs in the test set, since no information is available for prediction. In Table 4, we thus extended the G1 model by adding “auxiliary relations” to provide alternative sources of information from which the model can infer latent representations of protein–drug pairs that are not present in the training set DTI prediction task. We refer to these models as G* (see Section 2 and Fig. 1C).

**Table 4.** Performance obtained by incrementally extending the G1 model with one additional relation (task) at a time (G* model), with the stringent pair-stratification 5-fold CV on the LHU dataset.

Model	AUC	AUPR	Precision	Recall	# Relations
G1	0.501	0.538	0.100	0.222	1
G1 + Drug sim (G*)	0.723	0.639	0.748	0.172	2
G* + Prot sim	0.656	0.693	0.752	0.303	3
G* + Pfam (as relation)	0.751	0.701	0.758	0.504	4
G* + Pfam (as side)	0.763	0.743	0.786	0.764	3 (+1 side)

Table 4 shows that adding the drug–drug similarity computed with RDkit as self-relation (second row of Table 4) already improves all the evaluation metrics, since our two-relation model can now learn useful similarities between drugs instances even without observing them involved in drug–protein positive or negative pairs in the training data for the DTI prediction task. Subsequently adding the protein similarity as self-relation of the protein entity slightly decreases the AUC, but increases slightly the AUPRC and the recall.

When building data fusion models on ER graphs with NXTfusion, each additional source of information can be added as relation between entities or as “side information,” namely as conventional ML features. Depending on the type, the sparsity of the data, and the kind of information that we want to add, one of the two options might be optimal in each case. The last two rows of Table 4 show the difference in performance when the Pfam-domain information is added as additional protein–domain relation (see Fig. 1C), or as side information (mixed to the protein embeddings with a bilinear layer). In the first case, the new relation increases the performance by 4% respect to the G* without protein similarity and by 12% respect to the previous row. In the second case, the side information gives an additional 2% increase in AUC and an increase of 33% in Recall.

Table 4 shows that adding auxiliary relations to the ER graph factorized by NXTfusion indeed provides information that could be orthogonal to the one contained in the main relation. The final G* model has indeed an AUC 52% higher than the random result obtained by G1. Removing from the final G* graph the protein similarity (second row in Table 4), which locally lowers the AUC, produces a final AUC and AUPRC scores of 0.74 and 0.71, which are lower than the final G* model including it (last two rows).

### 3.4 CV stratification is key to avoid overestimated performance also on the YUNAN dataset

To extend the validation of our ER data fusion approach for DTI prediction beyond the LHU and LCE datasets, we retrieved the dataset used to validate the DTINet predictor (Luo *et al.* 2017). We reproduced the validation described by the authors, which is a randomized 10-fold CV. We refer to this dataset as YUNAN. It contains 1923 known DTIs (positive interactions), and we sampled 1923 putative negative interactions by randomly pairing proteins and drugs (see Section 2 for more details).

In the first rows of Table 5, we show the AUC and AUPRC scores of the DTInet method, presented in Luo *et al.* (2017), and other state-of-the-art approaches, such as BLMNII (Mei *et al.* 2013), NetLapRLS (Xia *et al.* 2010), HNM (Wang *et al.* 2014), and CMF (Zheng *et al.* 2013). The lower part of the table show the effect of incrementally adding relations to our initial G1 baseline ER graph (see Fig. 1D).

**Table 5.** Comparison on the YUNAN dataset between the AUC and AUPRC scores obtained by the DTInet model presented in Luo *et al.*(2017) (first row) and different G1 and G* ER-graphs^a^.

Model	AUC	AUPRC	# Relations
BLMNII	0.69	0.75	
NetLapRLS	0.83	0.88	
HNM	0.86	0.88	
CMF	0.86	0.87	
DTInet	0.91	0.93	

G1	0.84	0.80	1
G1 + protein similarity	0.89	0.86	2
G* + drug similarity	0.88	0.86	3
G* + PPI network	0.87	0.87	4
G* + DDI network	0.85	0.79	5
G* + drug–disease	0.86	0.87	5 (+1 side)
G* + protein–disease	0.86	0.83	5 (+1 side)

a For the nonincremental case see Supplementary Table S8.

In this dataset, adding a protein–protein self-relation describing protein similarities improves both AUC and AUPRC (respectively +6% and +7%), but the further addition of other entities does not increase the scores further. Due to the large size of the drug–disease and protein–disease relations, and their limited contribution to the prediction, we tested them one at a time and not together, as indicated by the number of relations in the last two rows of Table 5.

As observed also in Luo *et al.* (2017), the apparent early saturation of the performance shown in Table 5 might be caused by the fact that the information brought by the auxiliary relations are already “leaking” to the model because of the presence of similar drugs and proteins in the training and test sets during CV, resulting in a overestimation of the performance, similarly to what we observed in the Liu *et al.* (2015) dataset.

We thus followed the lead of DTINet authors and we benchmarked our approach on several variants of the YUNAN dataset (Luo *et al.* 2017). In each of them, we controlled some aspects that could lead to information leakage between training and testing folds, including

1. Limiting the Sequence Identity among proteins to 40%.
2. Removing drugs with Tanimoto similarity >60%.
3. Removing drugs with similar side effects (Jaccard score over 60%).
4. Removing drug pairs associated to similar diseases (Jaccard score over 60%).
5. Reducing both the similarity among proteins and drugs (combining the first two items).

These ablations on the initial YUNAN dataset, containing 1923 positive samples, reduce the number of positive DTIs respectively to 1332, 1268, 1265, 1077, and 900 cases. Negative DTIs were sampled in each case to have a 1:1 proportion between positive and negative labels.

Table 6 shows the results obtained in the most stringent settings, where the similarity of both drugs and proteins is reduced (Item 5 in the previous list). The performance obtained on the other dataset ablation experiments on YUNAN is shown in Supplementary Tables S13–S16. A summary of the comparison is shown in Supplementary Figs S1 and S2. Additional stratifications are shown in Supplementary Tables S9–S12.

**Table 6.** Comparison of DTInet and other state-of-the-art methods with our G* models on the YUNAN dataset after simulating more stringent prediction settings in which similar proteins and drugs have been removed (dataset ablation).

Model	AUC	AUPR	# Relations
BLMNII	0.61	0.69	
NetLapRLS	0.75	0.82	
HNM	0.74	0.79	
CMF	0.77	0.78	
DTInet	0.82	0.87	

G1	0.85	0.84	1
G1 + protein similarity	0.87	0.89	2
G* + drug similarity	0.88	0.91	3
G* + PPI network	0.87	0.88	4
G* + DDI network	0.88	0.87	5
G* + drug–disease	0.86	0.87	5 (+ 1 side)
G* + protein–disease	0.84	0.80	5 (+ 1 side)

By comparing the results before (Table 5) and after (Table 6) the stratification, we can see that all the models in the upper part of the table experience a significant decrease of their performance (−10% in AUC for DTInet). By contrast, our model performance on this more difficult dataset is quite robust. Adding the auxiliary relations contextualizing the similarity among proteins and drugs now shows their positive impact. Nevertheless, from Table 6, it appears that adding additional relations, such as PPI networks, DDI networks, drug–disease and protein–disease associations, still do not increase performance further. This might be due to the fact that, since this kind of data fusion models are based on learning similarities between instances of the entities, the first two relations are sufficient to provide all the information that the model can use for prediction on this dataset.

### 3.5 Data fusion for the prediction of drug–kinase affinity

We performed the last two benchmarks in this study on the DAVIS (Davis *et al.* 2011) and KiBA (Tang *et al.* 2014) datasets. These datasets are substantially different from the ones used so far because (i) they are specific to the kinase family of proteins and (ii) the main task is the regression of the real-valued affinity between kinases and drugs. While these settings are not likely to be optimal for our data fusion methods, since they focus on a specific protein family, we included this scenario to showcase the flexibility of our approach, which can tackle different prediction tasks by just changing the loss function used and the data loaded in the underlying ER graph, yielding comparable or just slightly lower performances with respect to specialized approaches.

To evaluate the performance, we thus used the MSE between predictions and experimental affinity values and the Concordance Index (CI) proposed in Öztürk *et al.* (2018). We reproduced the same 5-fold CV used in Öztürk *et al.* (2018) to directly compare our performance with the DeepDTA (Öztürk *et al.* 2018), KronRLS (Pahikkala *et al.* 2015), and SimBoost (He *et al.* 2017) methods. DeepDTA appears in three methodological variants. The CNN—PChem version uses Convolutional NNs (CNNs) to read the protein sequences, and Drug–drug similarity matrix from PubChem to describe the drugs (Öztürk *et al.* 2018). The SW—CNN version uses a sliding window to read the protein sequence and a CNN to read the Simplified Molecular-Input Line-Entry System (SMILES) description of the drugs (Öztürk *et al.* 2018). The best performing DeepDTA model (CNN—CNN) uses CNNs to read both protein sequences and SMILES drug descriptions.

The upper half of Table 7 shows the comparison between our G1 and G* data fusion models and DeepDTA, KronRLS, and SimBoost on the DAVIS dataset. The lower half shows the same comparison on the KiBA dataset. For a detailed analysis of the contribution of each entity in our ER graphs in different scenarios, see Supplementary Tables S17 and S18. On DAVIS, we obtain the best results with a G* model (see Fig. 1E) with two auxiliary relations contextualizing the similarities among protein and drug instances. It performs similarly to the CNN—CNN DeepDTA model in terms of MSE and slightly lower in terms of CI. With respect to G1, adding auxiliary relations decreases the MSE from 0.30 to 0.26.

**Table 7.** Comparison of our data fusion approach with the models benchmarked in Öztürk *et al.* (2018) on the DAVIS and KiBA datasets^a^.

Dataset	Model	CI	MSE	# Relations
DAVIS	KronRLS	0.87	0.38	
DAVIS	SimBoost	0.87	0.28	
DAVIS	DeepDTA—CNN—PChem	0.84	0.42	
DAVIS	DeepDTA—SW—CNN	0.89	0.42	
DAVIS	DeepDTA—CNN—CNN	0.88	0.26	

DAVIS	G1	0.86	0.30	1
DAVIS	G1 + drug similarity	0.85	0.29	2
DAVIS	G* + protein similarity	0.87	0.26	3
DAVIS	G* + Pfam (relation)	0.87	0.27	4
DAVIS	G* + Pfam (side)	0.86	0.27	3 (1 side)
DAVIS	G* + Morgan(side)	0.86	0.29	3 (2 side)

KiBA	KronRLS	0.78	0.41	
KiBA	SimBoost	0.84	0.22	
KiBA	DeepDTA—CNN—PChem	0.72	0.57	
KiBA	DeepDTA—SW—CNN	0.85	0.20	
KiBA	DeepDTA—CNN—CNN	0.86	0.19	

KiBA	G1	0.77	0.36	1
KiBA	G1 + drug similarity	0.80	0.30	2
KiBA	G* + protein similarity	0.80	0.29	3
KiBA	G* + Pfam (relation)	0.77	0.35	4
KiBA	G* + Pfam (side)	0.78	0.35	3 (1 side)
KiBA	G* + Morgan (side)	0.79	0.32	3 (2 side)

a Low MSE scores and high CI scores indicate the best predictions.

On the KiBA dataset, our G1 model performance is around 10% worse than the CNN—CNN DeepDTA model in terms of CI and significantly worse in terms of MSE. Adding relations to our G* model (see Fig. 1E) improves our performance, but we remain around 7% lower in terms of CI.

From Table 7, we can see that our G* models outperform the versions of DeepDTA that use the PChem drug–drug similarity matrix, but DeepDTA performance drastically improves when more detailed protein and drug sequence information is provided (i.e. CNN—CNN DeepDTA). This indicates that for kinase-specific methods is crucial to access more detailed information regarding the protein and the drug molecular characteristics with respect to our ER graph approach, in which proteins and drugs sequences are never explicitly considered by our model.

We then tried to mitigate this problem by adding as sparse side information in our model 30 000-dimensional Morgan fingerprints (Morgan 1965) describing the molecular structure of each drug (see Fig. 1E). As shown in the last row of Table 7, this did not improve performance on DAVIS, and provided just a slight improvement on KiBA.

Additionally, in Supplementary Table S19, we compare the performance of our model with the IDG-DREAM Drug-Kinase Binding Prediction Challenge (Cichońska *et al.* 2021).

## 4 Discussion

In this article, we used the NXTfusion (Raimondi *et al.* 2021) data fusion library to build several models for DTI prediction. NXTfusion extends the conventional MF paradigm by allowing nonlinear inference over an arbitrary number of data matrices (Relations between Entities) and side information. To do so, the data belonging to the domain of interest and their connections are gathered by the user and organized as an abstract ER graph, on which inference is performed. Data fusion is achieved by jointly training a multitask NN model able to reconstruct all the relations in the ER-graph. NXTfusion uses both its multitask approach and the side information to avoid the conventional “transductive” limitations of MF methods, which cannot generalize to unseen data (i.e. new rows or columns) without retraining (Zhang and Chen 2019).

We empirically showed that performing data fusion over heterogeneous sources of complementary information is helpful in real-life DTI scenarios in which little to no information is available for unknown or poorly known drugs or proteins. While the improvement of NXTfusion over other more specialized models for DTI binary prediction is sometimes limited, a key result of this study is that thanks to the addition of the auxiliary tasks, our model performances are robust when the prediction problem becomes increasingly more difficult, e.g. due more stringent protein and drug-based CV stratifications (Pahikkala *et al.* 2015) simulating real-life situations in which predictions are required for previously unseen drug and protein pairs (see Tables 4 and 6 and Supplementary Figs S1 and S2).

## Supplementary Material

btad348_Supplementary_Data

## Data Availability

The data used in this paper are publicly available from the corresponding publications. The code is freely available in our git repositories.
