# Supplementary material for: Nonlinear data fusion over Entity–Relation graphs for Drug–Target Interaction prediction
Source: Bioinformatics. 2023 May 31;39(6):btad348. doi: 10.1093/bioinformatics/btad348 (PMC10265447; doi:10.1093/bioinformatics/btad348)
Supplement: btad348_Supplementary_Data [file btad348_supplementary_data.pdf]

Supplementary Material

E. Mazzone, Y. Moreau, P. Fariselli, D. Raimondi

April 27, 2023

S1 Results on LHU dataset

In this section we show the supplementary results obtained on LHU datasets. We report the results used to determine the entities and relations in the optimal G* graphs. The last table show entity distribution over interaction classes.

S1.1 Cross validation with random stratification

Model Graph	AUC	AUPRC	Precision	Recall
G1	0.987	0.988	0.984	0.895
G1+Drug Sim.	0.988	0.980	0.990	0.891
G1+Prot. Sim.	0.987	0.989	0.982	0.920
G1+Pfam(main)	0.982	0.985	0.957	0.918
G1+Pfam(side)	0.947	0.943	0.996	0.704

Table S1: Table showing the performance of the model G* on the LHU dataset. Each row shows the effect of adding a single additional relation to the standard G1 model. Cross validation folds are randomly split.

Model Graph	AUC	AUPRC	Precision	Recall	# relations
G1	0.987	0.989	0.974	0.929	1
G1+Drug Sim.	0.989	0.990	0.991	0.906	2
G*+Prot. Sim.	0.983	0.986	0.964	0.920	3
G*+Pfam(main)	0.985	0.988	0.994	0.864	4

Table S2: Table showing the performance of the model G1 on the LHU dataset. Each row shows the effect of adding a single additional relation to the model G1/G* in the previous row (incremental). Cross validation folds are randomly split.

S1.2 Cross validation stratified per protein

Model Graph	AUC	AUPRC	Precision	Recall
G1	0.948	0.966	0.998	0.696
G1+Drug Sim.	0.954	0.962	0.981	0.600
G1+Prot. Sim.	0.947	0.938	0.998	0.688
G1+Pfam(main)	0.915	0.937	0.995	0.546
G1+Pfam(side)	0.903	0.884	0.996	0.500

Table S3: Table showing the performance of the model G1 on the LHU dataset. Each row shows the effect of adding a single additional relation to the standard G1 model (not incremental). Cross validation folds are split by protein sequence identity.

Model Graph	AUC	AUPRC	Precision	Recall	# relations
G1	0.935	0.919	0.996	0.641	1
G*+Drug Sim.	0.940	0.957	0.977	0.646	2
G*+Prot. Sim.	0.960	0.972	0.964	0.828	3
G*+Pfam(main)	0.939	0.958	0.978	0.769	4

Table S4: Table showing the performance of the model G1 on the LHU dataset. Each row shows the effect of adding a single additional relation to the model G1/G* in the previous row (incremental). Cross validation folds are split by protein sequence identity.

S1.3 Cross validation stratified per drug

Model Graph	AUC	AUPRC	Precision	Recall
G1	0.845	0.850	0.920	0.631
G1+Drug Sim.	0.855	0.889	0.950	0.530
G1+Prot. Sim.	0.879	0.885	0.935	0.613
G1+Pfam(main)	0.830	0.850	0.751	0.717
G1+Pfam(side)	0.870	0.880	0.962	0.501

Table S5: Table showing the performance of the model G1 on the LHU dataset. Each row shows the effect of adding a single additional relation to the standard G1 model (not incremental). Cross validation folds are split by drug SMILE.

Model Graph	AUC	AUPRC	Precision	Recall	# relations
G1	0.861	0.895	0.929	0.603	1
G1+Drug Sim.	0.888	0.904	0.910	0.736	2
G*+Prot. Sim.	0.829	0.849	0.842	0.711	3
G*+Pfam(main)	0.853	0.875	0.869	0.678	4

Table S6: Table showing the performance of the model G1 on the LHU dataset. Each row shows the effect of adding a single additional relation to the model G1/G* in the previous row (incremental). Cross validation folds are split by drug SMILE.

Entity	# in both classes	# of only non-interacting	# of only interacting	# Total
Proteins	271	1167	563	2001
Drugs	17	1547	1162	2726

Table S7: Summary of number of entity for each classes, we can see that the majority of them belong to only one group (0/1)

S2 Supplementary results on YUNAN dataset

Here we show the supplementary tables computed on YUNAN dataset.

Model Graph	AUC	AUPRC	Precision	Recall
G1	0.885	0.866	0.714	0.918
G1+Prot. Sim.	0.839	0.843	0.823	0.738
G1+Drug. Sim.	0.842	0.861	0.794	0.827
G1+Prot Net	0.854	0.841	0.770	0.866
G1+Drug Net	0.867	0.860	0.828	0.837
G1+Drug Dis	0.853	0.842	0.789	0.843
G1+Prot Dis	0.853	0.827	0.748	0.863

Table S8: Table showing the performance of the model G1 on the YUNAN dataset. Each row shows the effect of adding a single additional relation to the standard G1 model (not incremental). Cross validation folds are randomly split.

S2.1 Results of the different stratifications

Model Graph	AUC	AUPRC
G1	0.64	0.63
G1+Prot. Sim.	0.66	0.64
G*+Drug. Sim.	0.55	0.55
G*+Prot Net	0.64	0.63
G*+Drug Net	0.61	0.58
G*+Drug Dis	0.53	0.51
G*+Prot Dis	0.60	0.62

Table S9: Table showing the performance of the model G1 on the YUNAN dataset. Each row shows the effect of adding a single additional relation to the model G1/G* in the previous row (incremental). Cross validation folds are split by protein ID.

Model Graph	AUC	AUPRC
G1	0.86	0.86
G1+Prot. Sim.	0.85	0.84
G*+Drug. Sim.	0.83	0.82
G*+Prot Net	0.85	0.85
G*+Drug Net	0.79	0.82
G*+Drug Dis	0.77	0.78
G*+Prot Dis	0.82	0.82

Table S10: Table showing the performance of the model G1 on the YUNAN dataset. Each row shows the effect of adding a single additional relation to the model G1/G* in the previous row (incremental). Cross validation folds are split by drug ID.

Model Graph	AUC	AUPRC
G1	0.50	0.49
G1+Prot. Sim.	0.53	0.55
G*+Drug. Sim.	0.49	0.50
G*+Prot Net	0.56	0.56
G*+Drug Net	0.63	0.63
G*+Drug Dis	0.59	0.56
G*+Prot Dis	0.57	0.55

Table S11: Table showing the performance of the model G1 on the YUNAN dataset. Each row shows the effect of adding a single additional relation to the model G1/G* in the previous row (incremental). Cross validation folds are split by both protein and drug IDs.

Model Graph	AUC	AUPR
Random	0.84	0.83
(1)Unknown Protein	0.66	0.63
(2)Unknown Compound	0.85	0.85
(3)Unknown Couple	0.49	0.49

Table S12: Comparison between various stratification strategy over YUNAN dataset using the G1 model

Model Graph	AUC	AUPR
BLMNII	0.60	0.69
NetLapRLS	0.82	0.87
HNM	0.84	0.87
CMF	0.78	0.79
DTInet	0.87	0.90
main task	0.89	0.85
+protein similarity	0.88	0.88
+drug similarity	0.86	0.80
+prot net	0.89	0.89
+drug net	0.86	0.81
+ drug-disease	0.88	0.88
+ prot-disease	0.87	0.88
prot net, drug sim, drug side	0.90	0.90

Table S13: Table showing the performance of the model G1 on the YUNAN dataset deprived of Homologous Protein. Each row shows the effect of adding a single additional relation to the model G1/G* in the previous row (incremental). Cross validation folds are randomly split.

Model Graph	AUC	AUPR
BLMNII	0.63	0.71
NetLapRLS	0.74	0.81
HNM	0.76	0.80
CMF	0.84	0.84
DTInet	0.89	0.91
main task	0.89	0.86
+protein similarity	0.86	0.77
+drug similarity	0.87	0.89
+prot net	0.82	0.76
+drug net	0.79	0.80
+ drug-disease	0.83	0.84
+ prot-disease	0.82	0.84
prot net, drug sim, drug side	0.88	0.90

Table S14: Table showing the performance of the model G1 on the YUNAN dataset deprived of Similar Drugs. Each row shows the effect of adding a single additional relation to the model G1/G* in the previous row (incremental). Cross validation folds are randomly split.

Model Graph	AUC	AUPR
BLMNII	0.63	0.72
NetLapRLS	0.83	0.87
HNM	0.80	0.84
CMF	0.79	0.81
DTInet	0.89	0.92
main task	0.95	0.95
+protein similarity	0.96	0.97
+drug similarity	0.98	0.98
+prot net	0.94	0.95
+drug net	0.97	0.94
+ drug-disease	0.90	0.93
+ prot-disease	0.94	0.93

Table S15: Table showing the performance of the model G1 on the YUNAN dataset deprived of Drugs/Proteins associated with Similar Disease. Each row shows the effect of adding a single additional relation to the model G1/G* in the previous row (incremental). Cross validation folds are randomly split.

Model Graph	AUC	AUPR
BLMNII	0.62	0.72
NetLapRLS	0.80	0.86
HNM	0.80	0.84
CMF	0.78	0.78
DTInet	0.89	0.91
main task	0.83	0.80
+protein similarity	0.88	0.86
+drug similarity	0.83	0.80
+prot net	0.89	0.88
+drug net	0.88	0.86
+ drug-disease	0.86	0.81
+ prot-disease	0.85	0.83

Table S16: Table showing the performance of the model G1 on the YUNAN dataset deprived of Drugs associated with similar Side Effects. Each row shows the effect of adding a single additional relation to the model G1/G* in the previous row (incremental). Cross validation folds are randomly split.

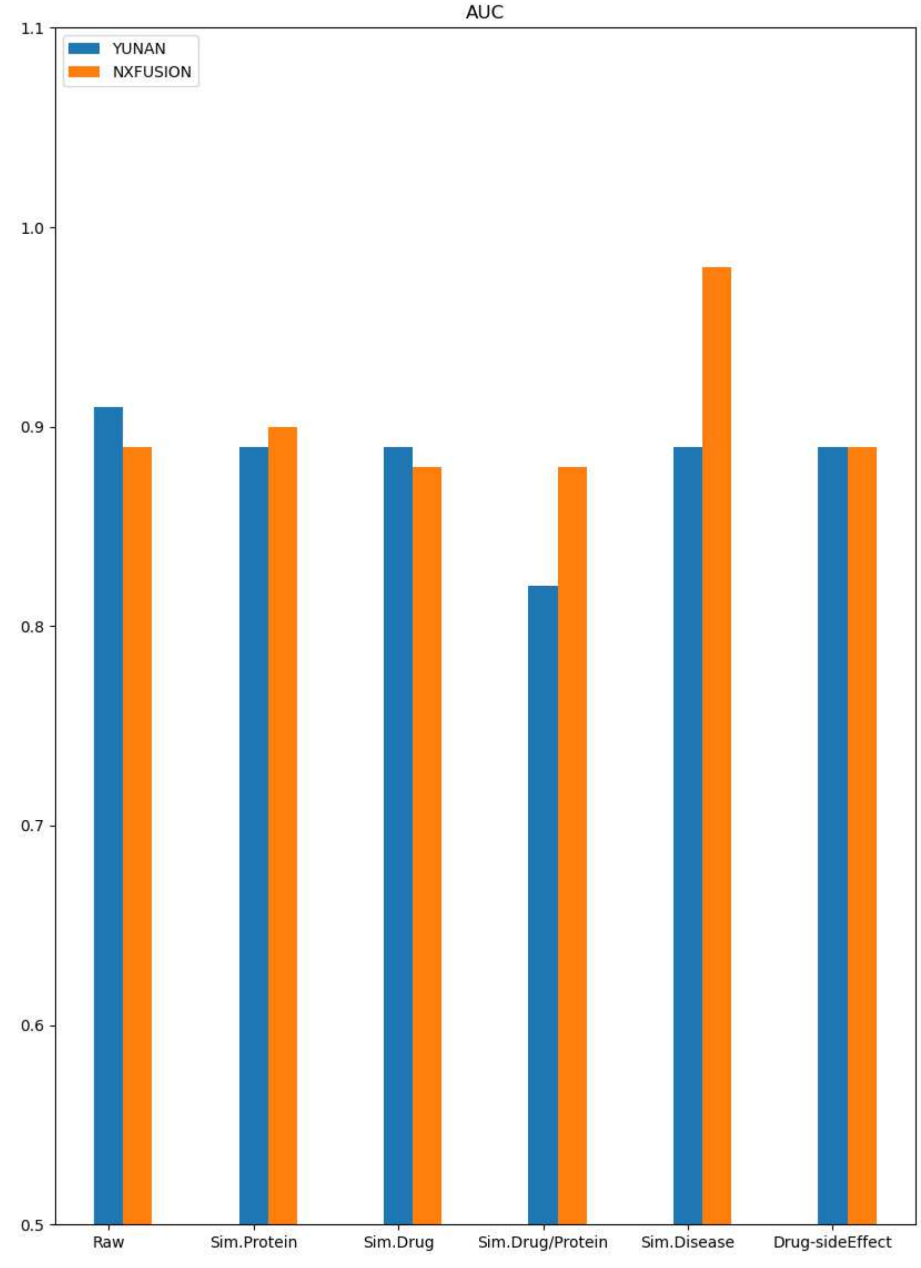

Figure S1: AUC Comparison between our best model (orange) and the results reported in [2] (blue), benchmarked against all the variations of the YUNAN dataset they proposed. Where 'Raw' means as no-changes, and other other characteristic are the one "hidden" in that particular setting.

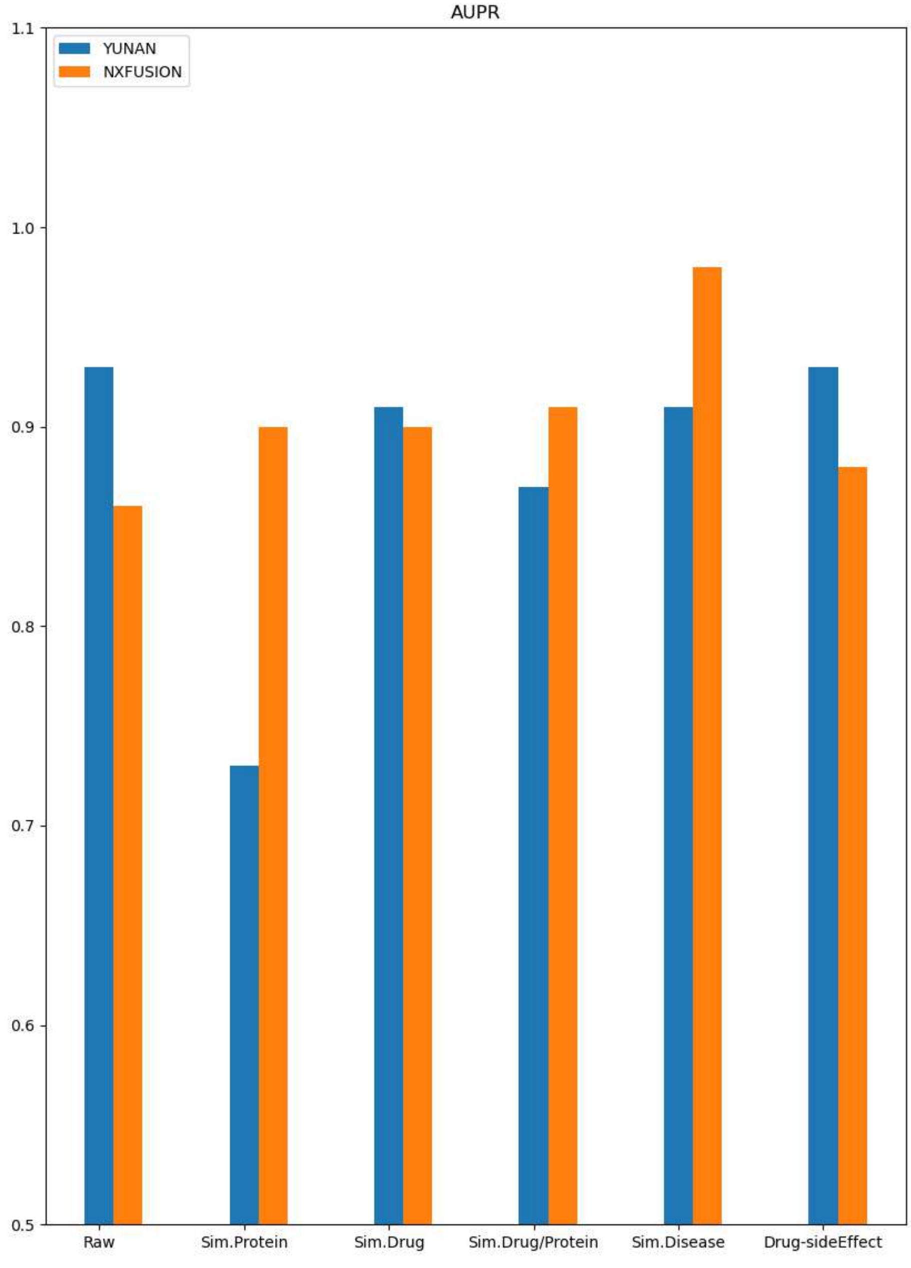

Figure S2: AUPR Comparison between our best model (orange) and the results reported in [2] (blue), benchmarked against all the variations of the YUNAN dataset they proposed. Where 'Raw' means as no-changes, and other other characteristic are the one hidden in that particular setting.

S3 Supplementary results on Davis and KiBA

Her we show supplementary tables on DAVIS and KiBA datasets. We show the results obtained in the binary Model Graphs, where a default threshold was chosen to binarize the prediction labels.

S3.1 Regression Problem

Model Graph	Ci	MSE
DeepDTA-CNN—PChem	0.84	0.42
DeepDTA-SW—CNN	0.89	0.42
DeepDTA-CNN—CNN	0.88	0.26
G1	0.846	0.330
G1+Drug Sim.	0.856	0.317
G1+Prot. Sim.	0.866	0.271
G1+Pfam(main)	0.852	0.296
G1+Pfam(side)	0.854	0.301

Table S17: Table showing the performance of the model G1 on the DAVIS dataset. Each row shows the effect of adding a single additional relation to the standard G1 model (not incremental). Cross validation folds are randomly split.

Model Graph	Ci	MSE
DeepDTA-CNN—PChem	0.72	0.57
DeepDTA-SW—CNN	0.85	0.20
DeepDTA-CNN—CNN	0.86	0.19
G1	0.779	0.354
G1+Drug Sim.	0.776	0.354
G1+Prot. Sim.	0.772	0.361
G1+Pfam(main)	0.800	0.299
G1+Pfam(side)	0.771	0.363

Table S18: Table showing the performance of the model G1 on the KiBA dataset. Each row shows the effect of adding a single additional relation to the standard G1 model (not incremental). Cross validation folds are randomly split.

S4 IDG-DREAM Drug-Kinase Binding Prediction Challenge

Here we show the performance of our approach on the IDG-DREAM Drug-Kinase Binding Prediction Challenge [1]. We trained different versions of our model for 60 epochs on Round1 data and then we tested on the Round2 data provided by the challenge organizers. We tried 4 different models, incrementally adding the following data to our ER-graphs in the following order: Drug Similarity (DS), Protein Similarity (PS) and 30000 dimensional Morgan Fingerprints (FP) as side information. G1 represents the base model in which only the protein-drug relation is factorized. We computed protein similarity with the all-against-all BLAST bitscore. We computed drug similairies and Morgan Fingerprints with RDkit. We compared our results with the official benchmark in the following table [1].

Model Graph	Spearman coeff.	RMSE
Q.E.D	0.532	0.962
Gregory Koytiger	0.527	0.897
AI Winter is Coming	0.515	0.939
DMIS_DK	0.515	0.970
hulab	0.477	1.248
METU_EMBL-EBI_CROssBAR	0.412	1.066
Baseline	0.400	1.123
thinng	0.394	1.146
KKT	0.363	1.116
siramshettyv2	0.358	0.994
ML-Med	0.353	1.190
Let_Data_Talk	0.330	1.372
oselot	0.307	1.113
Prospectors	0.300	1.196
Boun	0.286	1.156
KinaseHunter	0.270	1.325
AmsterdamUMC-KU-team	0.259	1.125
N121	0.224	1.287
**G1+DS+PS.**	0.217	1.18
ML-Med	0.213	1.154
Aydin	0.209	1.255
xuefeng	0.200	1.508
**G1+DS**	0.198	1.29
KERMIT-LAB	0.147	8.466
**G1+DS+PS+FP**	0.135	1.23
Druginase Learning	0.131	1.340
Prospectors	0.129	1.232
**G1**	0.117	1.38
MCIV	-0.023	1.170
CompBio-IGIB-kinase	-0.083	1534.124

Table S19: Table showing the performance of the model G1 on DREAM dataset. Each row shows the effect of adding a single additional relation to the standard G1 model (incremental).

References

[1] Anna Cichońska, Balaguru Ravikumar, Robert J Allaway, Fangping Wan, Sungjoon Park, Olexandr Isayev, Shuya Li, Michael Mason, Andrew Lamb, Ziaurrehman Tanoli, et al. Crowdsourced mapping of unexplored target space of kinase inhibitors. *Nature communications*, 12(1):3307, 2021.

[2] Yunan Luo, Xinbin Zhao, Jingtian Zhou, Jinling Yang, Yanqing Zhang, Wenhua Kuang, Jian Peng, Ligong Chen, and Jianyang Zeng. A network integration approach for drug-target interaction prediction and computational drug repositioning from heterogeneous information. *bioRxiv*, 2017.
